# Transitioning barium enema preparation protocols to CT colonography in the modern imaging era

**DOI:** 10.1007/s11604-025-01848-9

**Published:** 2025-08-09

**Authors:** Daisuke Tsurumaru, Yusuke Nishimuta, Katsuya Nanjo, Yutaro Shimomura, Kousei Ishigami

**Affiliations:** Department of Clinical Radiology, Graduate School of Medical Sciences, Kyushu University, 3-1-1 Maidashi, Higashi-ku, Fukuoka, Japan

**Keywords:** CT colonography, Colorectal cancer, Barium enema

## Abstract

**Purpose:**

To evaluate the feasibility of applying a barium enema-style bowel preparation protocol to CT colonography (CTC) in clinical practice.

**Materials and methods:**

11 patients underwent CTC using a simplified bowel preparation protocol based on magnesium citrate, similar to that used for barium enema. Two radiologists assessed the homogeneity of fluid tagging, volume of residual fluid, and degree of colonic distension in six colonic segments (cecum, ascending, transverse, descending, sigmoid, rectum) in both supine and prone positions. Each parameter was rated on a 4-point Likert scale (0 = optimal, 3 = inadequate). The mean score and proportion of segments achieving a score of 0 were calculated.

**Results:**

More than 85% of segments received a score of 0 for tagging uniformity and colonic distension, and over 90% for residual fluid. Mean scores were below 0.3 for all three parameters. These findings indicate that the preparation protocol provided sufficient colonic cleansing and distension comparable to previously reported standard protocols.

**Conclusion:**

A simplified bowel preparation protocol adapted from barium enema practice can be effectively applied to CT colonography, achieving clinically acceptable image quality.

## Introduction

Colorectal cancer (CRC) is one of the most common and deadly malignancies worldwide [1]. In Japan, CRC ranks second in cancer-related mortality among men and first among women [2]. Fecal immunochemical tests (FIT) are commonly used for screening, while colonoscopy and CT colonography (CTC) serve as diagnostic follow-up tools [3–6]. Historically, gastrointestinal contrast studies using barium sulfate were frequently employed for both screening and detailed examination. However, with the advancement of colonoscopy and CTC, contrast studies have become considered outdated, and their use has significantly declined. The European Society of Gastrointestinal and Abdominal Radiology (ESGAR) consensus guidelines also discourage the use of barium enema for colorectal cancer screening, further reinforcing this shift [7]. CTC has also become a widely accepted preoperative evaluation method for CRC in Japan, particularly using same-day protocols after colonoscopy [4]. Meanwhile, in facilities that continue to perform barium enemas, whether CTC can replace this role as a screening tool remains of interest. This technical note examines whether the workflow, including bowel preparation for barium enema, can be adapted to CTC.

## Materials and methods

This retrospective single-center study was approved by the institutional ethics committee. Informed consent was obtained from all patients prior to the examination. The study period was from April 2024 to March 2025. Patients included in this study were those who visited our hospital either for further evaluation following a positive FIT or for abdominal symptoms warranting colorectal examination, and who opted for CTC instead of colonoscopy.

### CTC protocol (Table 1)

**Table 1:** An example of schedule for CT colonography (Scan time: 9:00)

	Bowel preparation	Diet/Tagging
Two days before	22:00 – Sennoside A・B Calcium	
One day before	20:00 – Magcorol® 1 bottle 22:00 – sodium picosulfate	Low-residue meals + ColomforT Oral Supension® (barium sulfate) after each meal
Day of exam	Fasting 7:00 – bisacodyl suppository	

The protocol mainly followed our institution’s barium enema procedure, with the necessary addition of tagging for CTC. As low laxative, Sennoside A・B Calcium was taken 2 days before the examination to promote defecation. On the day before the exam, patients consumed low-residue meals and took a tagging agent (barium sulfate; ColomforT Oral Supension, Fushimi Pharmaceutical) immediately after each meal. That evening, they ingested 250 mL of Magnesium Citrate Oral Solution (Magcorol, Horii Pharmaceutical) and 5 ml of sodium picosulfate. On the day of the exam, patients fasted and received a bisacodyl suppository rectally in the morning. This preparation was a minor modification of our conventional barium enema protocol, with the sole addition of a tagging agent. The CT scan was performed using a 320-slice CT (Aquilion ONE/PRISM Edition, Canon Medical Systems) with the following parameters: tube voltage, 120 (kV), tube current (mA), automatic exposure control (SD = 10.5 mm), rotation time 0.5 (s/rot), pitch 0.8, and collimation 80-row*0.5 mm. Under the Japanese insurance system, CT equipment with 16 or more rows is required to claim CTC imaging fees. Intravenous contrast is not necessary. A rectal catheter is inserted, and carbon dioxide is insufflated using an automatic CO2 injector (PROTOCO2L; Bracco, Princeton, NJ) at approximately 20 mmHg. An antispasmodic agent, butylscopolamine, is administered intramuscularly just before scanning to suppress peristalsis and facilitate adequate colonic distension. After confirming sufficient colonic inflation on a pre-scan, scanning is performed in both supine and prone positions. If certain segments are inadequately distended, rescan with repositioning is permitted. The acquired image data are transferred to a workstation, where three-dimensional reconstructions, such as virtual endoscopy and barium enema-like views, are created. For clinical evaluation, the presence or absence of colonic lesions was investigated.

### CTC quality assessment

The quality of CTC was assessed by two radiologists in consensus using a 4-point scale to evaluate tagging uniformity, residual fluid, and colonic distension in both positions. The quantitative score was assessed according to a previously reported method based on a 4-point Likert scale [8]. Three quantitative parameters were evaluated: homogeneity of fluid tagging, volume of residual fluid, and degree of colonic distension (Table 2). For each parameter, a score ranging from 0 to 3 was assigned to each of the six colonic segments (cecum, ascending colon, transverse colon, descending colon, sigmoid colon, and rectum), separately for the supine and prone acquisitions. Scores from both positions were then combined, and for each colonic segment, two variables were calculated for each parameter: the number of segments with a score of 0, and the mean score [9].

**Table 2:** Likert scale used for the quantitative scores

	Score 0	Score 1	Score 2	Score 3
Homogeneity of fluid tagging	Homogeneous fluid tagging with uniform density and/or non-untagged solid residues	Homogeneous fluid tagging with uniform density or/and less than 5 unmarked solid residues	Partially homogeneous fluid tagging with layers of different densities and/or 5 to 10 unmarked solid residues	Absence of fluid tagging and/or more than 10 unmarked solid residues
Volume of residual fluid	< 25% of CT axial diameter	25–50% of CT axial diameter	50–75% of CT axial diameter	> 75% of CT axial diameter
Colon distension	> 75% of the maximum achievable	50–75% of the maximum achievable	25–50% of the maximum achievable	< 25% of the maximum achievable

## Results

During the study period, 11 patients (5 males and 6 females; median age, 62 years; range, 34–93) underwent CTC. The indications were: six for further evaluation of positive FIT, two for defecation-related discomfort, three for follow-up of prior colorectal polyps. Findings included colonic diverticula in three patients and a sigmoid colon polyp in one patient. The other seven patients had no abnormalities. CTC quality was sufficient for interpretation in all cases, and no complications related to the procedure were observed.

**Fig. 1:**
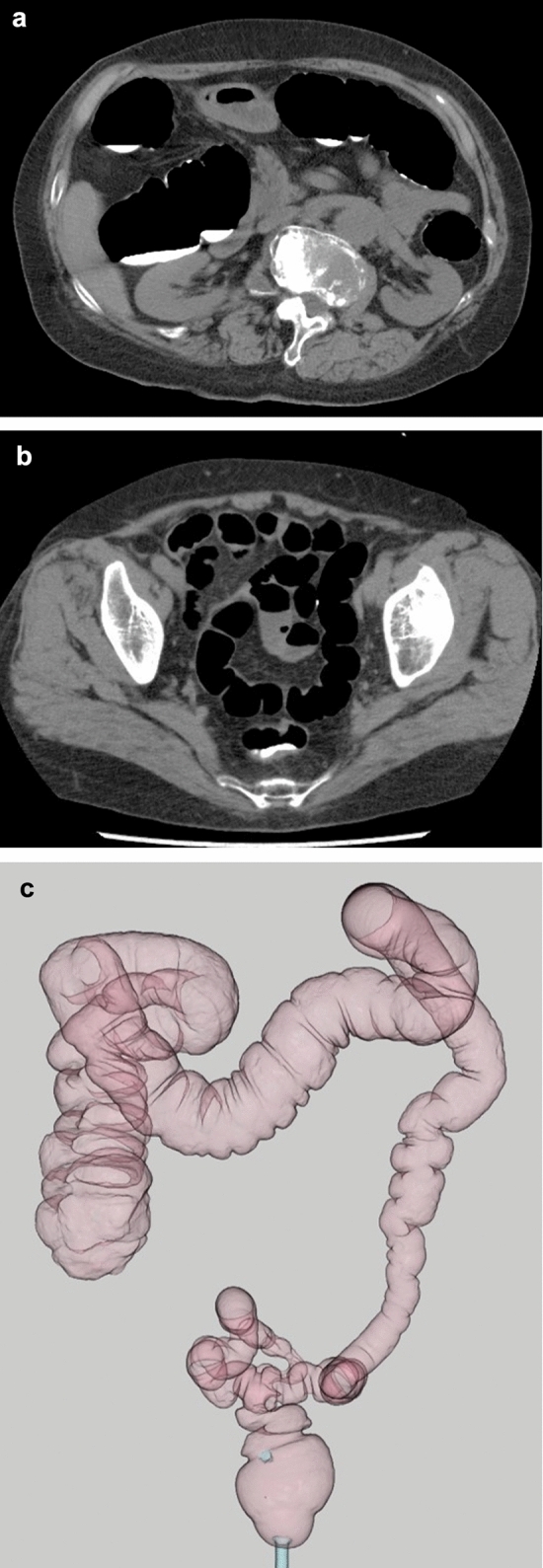
CT colonography in an 81-year-old woman undergoing evaluation for a positive fecal occult blood test. Supine position. **A**, **B** Axial CT images showing sufficient luminal distension, minimal residual fluid, and uniform tagging throughout the colon (score 0). In rectum (B), bowel distension is suboptimal (score 1) (arrow). **C** Barium enema-like view demonstrates generally good distension throughout the colon except for the upper rectum (arrow)

Quantitative assessment of bowel preparation is summarized in Table 3. Among the three parameters, residual fluid showed the highest score 0 rates, with 100% in the rectum and over 95% in all other segments. The mean score for residual fluid was consistently low, with 0.00 in the rectum and ≤ 0.18 in all segments. Colonic distension was generally adequate, with a score 0 rate exceeding 95% in most segments, except for the sigmoid colon (77.3%). Mean scores for distension ranged from 0.05 to 0.23. Fluid tagging showed slightly more variability, with score 0 observed in 77.3% to 86.4% of segments. The mean scores ranged from 0.18 to 0.32. Representative images of CTC with adequate fluid tagging and colonic distension are shown in Fig. 1.

**Table 3:** Quantification of bowel preparation quality

	Homogeneity of fluid tagging		Volume of residual fluids		Colon distension	
	Score 0	Mean	Score 0	Mean	Score 0	Mean
Rectum	18 (81.8%)	0.27	22 (100.0%)	0.00	21 (95.5%)	0.05
Sigmoid	19 (86.4%)	0.18	19 (86.4%)	0.18	17 (77.3%)	0.23
Descending	17 (77.3%)	0.32	21 (95.5%)	0.09	18 (81.8%)	0.18
Transverse	17 (77.3%)	0.27	19 (86.4%)	0.18	21 (95.5%)	0.05
Ascending	18 (81.8%)	0.27	21 (95.5%)	0.14	21 (95.5%)	0.14
Cecum	17 (77.3%)	0.32	21 (95.5%)	0.14	21 (95.5%)	0.14

## Discussion

In our cohort, score 0 rates exceeded 95% in most segments for residual fluid and distension, and exceeded 77% even for tagging, indicating that the preparation quality was clinically acceptable and comparable to that in previous studies [8]. These results suggest that our simplified protocol provides a sufficient level of bowel preparation for diagnostic CTC. The findings suggest that CTC can feasibly replace barium enema as a screening examination, using a similar procedural workflow. Bowel preparation methods for CTC generally fall into two categories: those modeled on colonoscopy and those derived from barium enema protocols. In recent years, the total volume of cleansing agents has tended to decrease, improving patient compliance [10]. In this study, we demonstrated that a slightly modified version of our traditional barium enema preparation—simply adding a tagging agent—was sufficient. Since tagging involves only minor additions to the previous day’s meals, the additional burden to the patient is minimal. As all procedures were conducted within the scope of the national health insurance system, there were no ethical concerns regarding this protocol.

Barium enema has historically been a widely used modality in Japan, but it is a technically demanding examination that typically requires the direct involvement of physicians. In contrast, CTC can be performed solely by radiologic technologists. From a healthcare resource perspective, this distinction highlights a key advantage of transitioning from barium enema to CTC. Such a protocol may therefore enhance the acceptance and implementation of CTC in clinical practice, supporting its replacement of barium enema in appropriate clinical settings. According to Hirofuji et al., low-dose CT colonography protocols can achieve radiation exposure levels lower than those of traditional barium enema [11]. This further supports the notion that transitioning from barium enema to CTC is justified from the perspective of radiation safety.

This study has several limitations, including a small sample size and a single-center retrospective design. A direct comparison between barium enema and CTC was not performed. Moreover, although a structured scoring system was used, the interpretation still involved a degree of subjectivity, and future studies should include quantitative evaluation.

In conclusion, this technical note demonstrates that the operational protocol used for barium enema can be adapted to CTC. In a clinical environment where barium enema is becoming obsolete, repurposing this workflow for CTC may offer a meaningful alternative in colorectal cancer screening.
